# The Development of Gonadotropins for Clinical Use in the Treatment of Infertility

**DOI:** 10.3389/fendo.2019.00429

**Published:** 2019-07-03

**Authors:** Bruno Lunenfeld, Wilma Bilger, Salvatore Longobardi, Veronica Alam, Thomas D'Hooghe, Sesh K. Sunkara

**Affiliations:** ^1^Faculty of Life Sciences, Bar-Ilan University, Ramat Gan, Israel; ^2^Medical Affairs Fertility, Endocrinology and General Medicine, Merck Serono GmbH, Darmstadt, Germany; ^3^Global Medical Affairs Fertility, Merck Healthcare KGaA, Darmstadt, Germany; ^4^Global Clinical Development, EMD Serono, Rockland, MA, United States; ^5^A Business of Merck KGaA, Darmstadt, Germany; ^6^Organ Systems, Group Biomedical Sciences, Department of Development and Regeneration, KU Leuven (University of Leuven), Leuven, Belgium; ^7^Department of Obstetrics and Gynecology, Yale University, New Haven, CT, United States; ^8^Assisted Conception Unit, King's College London, Guy's Hospital, London, United Kingdom

**Keywords:** recombinant gonadotropin, follicle stimulating hormone, luteinizing hormone, fertility, pregnancy, pre-clinical, clinical

## Abstract

The first commercially available gonadotropin product was a human chorionic gonadotropin (hCG) extract, followed by animal pituitary gonadotropin extracts. These extracts were effective, leading to the introduction of the two-step protocol, which involved ovarian stimulation using animal gonadotropins followed by ovulation triggering using hCG. However, ovarian response to animal gonadotropins was maintained for only a short period of time due to immune recognition. This prompted the development of human pituitary gonadotropins; however, supply problems, the risk for Creutzfeld–Jakob disease, and the advent of recombinant technology eventually led to the withdrawal of human pituitary gonadotropin from the market. Urinary human menopausal gonadotropin (hMG) preparations were also produced, with subsequent improvements in purification techniques enabling development of products with standardized proportions of follicle-stimulating hormone (FSH) and luteinizing hormone (LH) activity. In 1962 the first reported pregnancy following ovulation stimulation with hMG and ovulation induction with hCG was described, and this product was later established as part of the standard protocol for ART. Improvements in immunopurification techniques enabled the removal of LH from hMG preparations; however, unidentified urinary protein contaminants remained a problem. Subsequently, monoclonal FSH antibodies were used to produce a highly purified FSH preparation containing <0.1 IU of LH activity and <5% unidentified urinary proteins, enabling the formulation of smaller injection volumes that could be administered subcutaneously rather than intramuscularly. Ongoing issues with gonadotropins derived from urine donations, including batch-to-batch variability and a finite donor supply, were overcome by the development of recombinant gonadotropin products. The first recombinant human FSH molecules received marketing approvals in 1995 (follitropin alfa) and 1996 (follitropin beta). These had superior purity and a more homogenous glycosylation pattern compared with urinary or pituitary FSH. Subsequently recombinant versions of LH and hCG have been developed, and biosimilar versions of follitropin alfa have received marketing authorization. More recent developments include a recombinant FSH produced using a human cell line, and a long-acting FSH preparation. These state of the art products are administered subcutaneously via pen injection devices.

## Introduction

It was observed in 1927, by Ascheim and Zondek, that the blood and urine of pregnant women contained a gonad-stimulating substance, human chorionic gonadotropin (hCG) (1, 2). Seegar-Jones and colleagues demonstrated in the 1940s that hCG was produced by the placenta (3). In 1929, Zondek proposed, based on his experiments and those of Smith, that two hormones were produced by the pituitary gland, both of which stimulated the gonads (4–6). These hormones were described as gonadotropins and subsequently named follicle-stimulating hormone (FSH) and luteinizing hormone (LH), according to their specific actions. The biological activity of gonadotropins suggested that they might be useful for the treatment of patients who were infertile. These observations eventually led to the development of pure gonadotropin products that have enabled the birth of millions of children to people affected by infertility.

**Figure 1:**
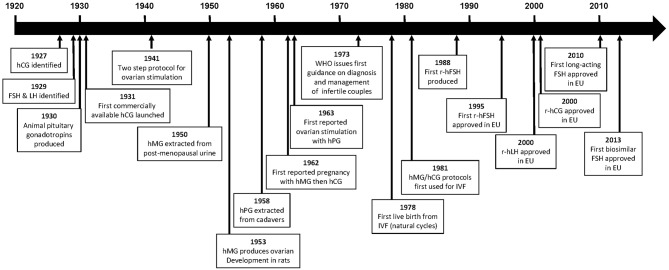
Time line of major events in the development of gonadotropins. CHO, Chinese hamster ovary; FSH, follicle-stimulating hormone; hCG, human chorionic gonadotropin; hMG, human menopausal gonadotropin; LH, luteinizing hormone; r-hCG, recombinant human chorionic gonadotropin; r-hFSH, recombinant human follicle-stimulating hormone; r-hLH, recombinant human luteinizing gonadotropin.

This review provides an overview of the major milestones in the development of gonadotropin products (Figure 1), as well as issues that may have affected decision making during the development processes, and summarizes the available evidence supporting the use of recombinant gonadotropin products for the treatment of infertility.

## Human Chorionic Gonadotropin

The first commercially available gonadotropin was an hCG extract launched by Organon in 1931 (4). However, the original product was of limited use owing to a lack of reproducibility, in part due to the use of animal units (mouse or rat) to measure bioactivity (7). Reproducibility was greatly improved in 1939 when the League of Nations developed the international standard for hCG; one International Unit (IU) of hCG was defined as the activity contained in 0.1 mg of the reference hCG preparation which was pooled from six sources (8). Following the introduction of this standard, purified hCG preparations extracted from the urine of women during the first half of pregnancy, with bioactivity up to 8,500 IU/mL, became available (9, 10).

### Clinical Use

In women, hCG is used during infertility treatment to trigger final follicular maturation and ovulation, as well as for luteal phase support. In men, it is used to stimulate production of testosterone by the Leydig cells in cases of hormone deficiency as well as in male hypogonadism.

## Animal Pituitary Gonadotropins

The first animal pituitary gonadotropin was swine pituitary gonadotropin [containing both FSH and LH (11, 12)], followed by hog and sheep pituitary extracts and pregnant mare serum gonadotropin (2, 4, 7, 13). With the availability of both placental and pituitary hormones, the two-step protocol for ovarian stimulation using an animal gonadotropin followed by final maturation and triggering with hCG, was introduced for women in 1941 by Mazer and Ravetz (2, 14). However, owing to their non-human origin, the ovarian response to animal gonadotropins was only maintained in women for a limited duration because of human–animal immune recognition (2, 15).

As a result of the limited clinical value of the animal gonadotropins, human pituitary gonadotropins extracted either post-mortem from human pituitaries or from the urine of postmenopausal women were investigated (2).

## Cadaveric Human Pituitary Gonadotropins

In 1958, Gemzell extracted FSH from pituitaries obtained from human cadavers and reported successful follicle development using this preparation, which was later given to women together with hCG to induce ovulation (16, 17). In 1963, ovarian stimulation with cadaveric human pituitary gonadotropin in hypophysectomised individuals was successfully performed by Bettendorf et al. (7). Owing to their source, these products were produced by several government agencies. Although used successfully for a number of years, these human pituitary preparations were discontinued in the 1980s because of supply problems and the risk for Creutzfeldt-Jakob disease that resulted from the source of these products (human cadavers) (2, 4, 18–20).

## Human Menopausal Gonadotropin

Human menopausal gonadotropin (hMG), which contains two gonadotropin components corresponding to the pituitary-related hormones, FSH and LH, was first successfully extracted from the urine of post-menopausal women in 1950. In 1953 hMG was shown to produce ovarian stimulation in female hypophysectomised infantile rats, and Leydig cell stimulation and full spermatogenesis in male hypophysectomised infantile rats (2, 4, 21). These experiments suggested that hMG would be useful in humans; however, to enable clinical testing, large-scale extraction and purification methods were required, in addition to an agreed standard to enable reproducibility. Furthermore, the starting dose for humans needed to be established. The first hMG preparations were registered by Serono in Italy in 1950, but these were impure in terms of protein content and did not have standardized proportions of FSH and LH. Subsequent preparations contained equal proportions of FSH and LH (for example, Pergonal 75 contained 75 IU FSH and 75 IU LH), in addition to other unwanted urinary proteins (2, 4). Additionally, the bioactivity of the first hMG preparations was measured in “animal” units (mouse or rat); a “rat unit” was the minimum amount of preparation required to induce oestrus in 28-day old female rats (“mouse units” were defined in a similar manner). The bioactivity, therefore, varied depending on the strain of animal used and a uniform standard was required to facilitate clinical use.

The first reference standard for hMG was based upon batches produced by kaolin extraction of menopausal urine (hMG 20, hMG 20a, and hMG 24) and provided by Organon Newhouse (2, 7). However, by 1959, most of the reference product had been used and further batches could not be provided. At this time, the Serono Institute in Rome offered 50 g of Pergonal 23 (containing equal proportions of FSH and LH) to act as the reference preparation and this material was subsequently used as the International Reference Preparation (IRP) (2, 7, 22). As well as facilitating greater reproducibility in clinical testing, the study of day-to-day variation of gonadotropins and steroid secretion during the normal menstrual cycle and during pregnancy was enabled by the availability of a reference product (2, 4, 7, 23). The aim of these studies was to understand the fundamental variability of gonadotropins in women so that these physiological concepts or patterns could be applied in future clinical tests.

Clinical trials were initiated and, in 1959, hMG (150 U/d for 4 days) was demonstrated to induce the expected, desirable changes in the endometrium and vaginal epithelium (24) and to induce steroid secretion, in women with anovulatory, hypogonadotropic hypopituitary and primary amenorrhea (2, 4, 22, 24–26). This was followed 3 years later by a report from Lunenfeld et al. of the first pregnancy in a patient with hypopituitary hypogonadotropic amenorrhea following ovulation induction with hMG and final oocyte maturation with hCG, no adverse events were reported for this pregnancy (26). This approach subsequently became the standard protocol for ovulation induction treatment of infertility (2, 26, 27).

The World Health Organization (WHO) Expert Committee of Biological Standardization defined, in 1972, the IU for both FSH and LH (then named interstitial cell stimulating hormone [ICSH]) as the respective activities contained in 0.2295 mg of the IRP of hMG (28). The use of IU depends upon determination of the linearity of the bioactivity of the gonadotropin product. The bioactivity of FSH, for example, is determined by the Steelman–Pohley bioassay. This bioassay is based on comparison between the test FSH preparation and the international reference standard (defined by the WHO) of FSH-induced augmentation of ovarian weight in immature female rats co-treated with a high dose of hCG (29). One year later, in 1973, the WHO issued their first guidance on the diagnosis and management of infertile couples, recommending an effective daily dose of 150–225 IU hMG for hypogonadotropic patients (WHO Group I), and 75–150 IU for anovulatory normogonadotropic patients (WHO Group II) (24, 26, 30).

Steptoe and Edwards pioneered *in vitro* fertilization (IVF) procedures using natural cycles, achieving the first live birth in 1978 (31); one pregnancy was reported following 101 attempts (32). However, in 1981, Jones and Jones established hMG/hCG protocols as described by Lunenfeld et al. (26) as the standard approach for ovarian stimulation in assisted reproductive technologies (ART), achieving one pregnancy after three attempts (33, 34). These protocols were later revised when the outcome of ovarian stimulation in ART treatment changed from mono-follicular to multi-follicular development (4).

Improvements in purification techniques enabled the development of an hMG preparation with fewer impurities. However, these extraction steps also removed LH activity (22) and hCG had to be added to re-establish the FSH:LH ratio, resulting in highly purified hMG containing approximately 30% identified impurities that varied from batch to batch (2). Polyclonal immunopurification techniques also resulted in an FSH preparation devoid of LH activity (35). However, this preparation still contained many unwanted urinary proteins. The development of monoclonal FSH antibodies to replace the polyclonal antibodies allowed greater purification of urinary products resulting in a highly purified FSH preparation (Metrodin HP [EU]; Fertinex [USA]; highly purified urofollitropin) containing about 9000 IU of FSH per mg of protein, <0.1 IU LH activity and <5% unidentified urinary proteins. This enabled the formulation of smaller injection volumes and subcutaneous, rather than intramuscular, administration (2). The currently available hMG preparations are considered safe with the most common adverse events, as reported by clinical trials, being ovarian hyperstimulation, abdominal pain, headache, enlarged abdomen, inflammation at the injection site, pain at the injection site and nausea; the incidence rate of these events was 2–7% (36).

Despite these advances in the preparation of urinary gonadotropin products, supplies were limited owing to the finite donor supply, and batch-to-batch variability was an issue because of the source (2). These issues were overcome by the development of recombinant human FSH (r-hFSH) and subsequently recombinant human LH (r-hLH) and hCG (r-hCG) (2).

### Clinical Use

hMG is approved for development of a single Graafian follicle in women with anovulation and multifollicular development in women undergoing controlled ovarian hyperstimulation as part of ART treatment. hMG has also been demonstrated to be effective for the induction or restoration of secondary sexual development and fertility due to androgen deficiency in males with hypogonadotropic hypogonadism, when used in combination with hCG (37–39).

## Recombinant Gonadotropins

Recombinant biological products are proteins produced using recombinant DNA technology that utilize biological processes to produce large molecule drugs that cannot be produced using synthetic chemistry. Recombinant gonadotropins were developed to avoid the limitations inherent to the earlier urine-derived gonadotropin products, since recombinant products can be produced in large volumes with high purity and without variability in composition. As with hMG, the recombinant products can be used for the treatment of both male and female infertility.

### Follicle-Stimulating Hormone

There are currently three r-hFSH products on the market: follitropin alfa, follitropin beta, and follitropin delta. A fourth product, follitropin epsilon, has been reported as being in development (40). Follitropin alfa and follitropin beta are produced in Chinese hamster ovary (CHO) cell lines, whereas follitropin delta is produced in human fetal retinal cells; all these r-hFSH products have an amino acid sequence identical to that of endogenous human FSH. FSH has a relatively short biological half-life of about 1 day (40), necessitating daily administration. There has therefore been interest in long-acting formulations, and one such product is available, the long-acting r-hFSH analog corifollitropin alfa [elimination half-life: corifollitropin alfa, 70 (59–82) hours (41); follitropin alfa, terminal elimination half-life 24 h (42)].

### Follitropin Alfa and Follitropin Beta

The originator follitropin alfa (GONAL-f; Merck KGaA, Darmstadt, Germany) was first produced by Serono (a predecessor company of Merck KGaA) in 1988 and received a marketing license for clinical use in both women and men in the EU in 1995 (40) and in the USA in 2004 (42). Subsequently, two biosimilar versions of follitropin alfa have become available, for use in both women and men, Ovaleap (Teva B.V., Haarlem, the Netherlands), which received marketing authorization in Europe in 2013 (43), and Bemfola (Afolia, Finox Biotech AG, Balzers, Liechtenstein), which received marketing authorization in Europe in 2014 (44). The biosimilars are not currently approved in the USA. The safety profile of the biosimilar follitropin alfa preparations is similar to that of the originator product (43, 44). Follitropin beta (Puregon; Merck & Co., Kenilworth, NJ) received marketing authorization in Europe in 1996 and in the USA (Follistim AQ) in 2004 (45, 46). The risk/benefit balance of follitropins alfa and beta are considered positive, with the main adverse events reported being headache, ovarian cysts, local injection site reactions (e.g., pain, erythema, hematoma, swelling, and/or irritation at the site of injection) and mild or moderate OHSS (40, 43, 44).

Although both follitropin alfa and follitropin beta are produced in CHO cells, the vectors used for gene expression differ. Follitropin alfa is produced in CHO cell lines that have been transfected with separate expression vectors for the α- and β-FSH genes, the master cell cultures having been selected to co-amplify both genes (47), whereas follitropin beta (Puregon) is produced in CHO cell lines transfected with a single expression vector containing both α- and β-FSH genes (48). Culture processes also differ, in that large-scale culture of follitropin alfa occurs in a bioreactor, followed by purification of the culture supernatant by an ultrafiltration step and five chromatographic steps, with the main chromatographic purification step achieved through immunoaffinity (using a murine-derived anti-FSH monoclonal antibody) (47). Following large-scale culture of follitropin beta, r-hFSH is isolated from culture supernatant by a series of chromatographic steps including anion and cation exchange chromatography, hydrophobic interaction chromatography and size exclusion chromatography (48).

Owing to differences in the production and purification of follitropin alfa and follitropin beta there are differences in their glycosylation, and they have different sialic acid residue compositions and isoelectric coefficients. The isoelectric point band (pI) for follitropin alfa is narrower than that of follitropin beta (4–5 and 3.5–5.5, respectively), furthermore, follitropin alfa contains fewer isoforms with a pI <4 (9 and <24%, respectively) (49). These variations result in follitropin alfa being slightly more acidic and follitropin beta more basic, which influences their metabolic clearance, half-life (Table 1), and biological activity (48, 49, 54). The variance in mean specific FSH activity between follitropin alfa and follitropin beta (13,645 and 7,000–10,000 U/mg protein, respectively) affects the amount of protein required per injection (55). Follitropin alfa was originally dosed in IU based on its bioactivity in the Steelman-Pohley assay. However, owing to the consistency of the preparation it was possible to determine its specific activity, which is the ratio of the bioactivity (IU) to the protein content (mg, determined by size exclusion HPLC). Follitropin alfa can therefore be provided in injection devices filled-by-mass, which resulted in more consist ovarian response and reduced cycle cancelation rate, intra-cycle dose adjustment and repetitive monitoring (56, 57).

**Table 1:** Pharmacokinetics of a single dose of subcutaneous follitropin alfa 150 IU, follitropin beta 150 IU, follitropin delta (individualized dose), follitropin epsilon 150 IU and corifollitropin alfa in healthy women (46, 50–53).

**Mean value**	**Follitropin alfa**	**Follitropin beta**	**Follitropin delta**	**Follitropin epsilon**	**Corifollitropin alfa**
C_max_	3 IU/L	8 IU/L	–^a^	5.2 IU/L	4.2 ng/mL
t_max_ (h)	16	12	10	22	44
Bioavailability (%)	74	77	64	–	58
t_1/2β_ (h)	37	40^b^ (IM)	40	29	70
CL (L/h)	0.6^c^	0.01^d^	0.6	–	0.13

a *Value not reported but specified as being 1.4-fold higher than that of follitropin alfa (GONAL-f)*.

b *Measured after intramuscular administration*.

c *Measured after intravenous administration*.

d *Units are l/h/kg*.

*CL, clearance; C_max_, maximum plasma concentration; h, hours; t_1/2β_, terminal elimination half-life; t_max_, time to C_max_*.

Despite the disparities between follitropin alfa and follitropin beta, results of head-to-head clinical studies and retrospective studies comparing the two products for ovarian stimulation in women undergoing IVF have shown no significant differences between the preparations in terms of efficacy or safety (58–61). In the largest randomized prospective comparison, conducted in 172 women treated with follitropin alfa and 172 women treated with follitropin beta, a dose of 150 IU/day resulted in 13.0 and 12.4 oocytes obtained with each treatment (primary outcome), respectively, whereas at a dose of 300 IU/day, numbers were 6.1 and 7.1, respectively (60). Clinical pregnancy rates (secondary outcome) were similar with both preparations; 33.5% per cycle and 37.4% per embryo transfer with follitropin alfa 150 IU/day and 32.9% per cycle and 36.4% per embryo transfer with follitropin beta 150 IU/day (60).

The two biosimilar follitropin alfa products (Ovaleap and Bemfola) are considered to be similar to the reference product, GONAL-f; however, as a result of post-translational modifications, their structures are not identical. This is the result of differences in the processes used for their production and purification, including the cell line (despite all being produced using CHO cells) (43, 44). Specifically, differences in glycosylation were observed between the biosimilars and GONAL-f, with Bemfola showing higher antennarity, higher sialylation and higher batch-to-batch variability in activity compared with GONAL-f (62), whereas Ovaleap has a higher amount of the sialic acid N-glycolyl neuraminic acid compared with GONAL-f (63). For both biosimilars, the differences compared with GONAL-f were considered by regulatory agencies as minor and acceptable. Furthermore, a recent report on validation procedures for the Ovaleap manufacturing process showed the processes to be both robust and consistent, and that the resulting r-hFSH had similar characteristics to GONAL-f when molecular mass, primary structure, secondary structure, biological activity and product-related impurities were considered (64). Nevertheless, the observed differences may have a biological impact, including on FSH receptor activation, which has generated discussion regarding the potential clinical impact of these differences, particularly in “non-ideal” patients (i.e., older, poor or suboptimal responders or with worse prognosis factors), as by their nature there is always variation in biologics (65).

EMA guidelines recommend that, to determine clinical comparability, the efficacy of the reference and the similar biologic should be assessed in a randomized, parallel-group clinical trial, with number of oocytes retrieved as the primary endpoint, and ovarian hyperstimulation syndrome (OHSS) as an adverse reaction of special interest (66). Both Bemfola and Ovaleap have demonstrated equivalence to GONAL-f in terms of the number of oocytes retrieved (primary outcome) in women receiving ART (67, 68). Other outcomes (secondary outcomes), including pregnancy and live birth rate (LBR), have been reported as comparable to, or not statistically significantly different from, the originator product (GONAL-f) (69, 70).

A *post-hoc* pooled analysis of data obtained from randomized controlled trials (RCTs) indicated that treatment with GONAL-f is associated with a higher LBR (*p* = 0.037; primary endpoint) and lower OHSS (*p* = 0.011; secondary endpoint) than treatment with the biosimilars (Bemfola or Ovaleap) (69). However, further meta-analysis of data obtained from RCTs, ongoing post marketing real-world data studies and pharmacovigilance data concerning the use of these biosimilars are needed to ensure comparable clinical efficacy of these therapies to the originator in clinical practice.

### Follitropin Delta

Follitropin delta (Rekovelle; Ferring Pharmaceuticals, St. Prex, Switzerland) is produced using a human cell line, PER.C6 human fetal retinal cells, and received a marketing license in Europe in 2016 (51). It has a different glycosylation pattern from both follitropin alfa and follitropin beta (71). Follitropin delta has a higher proportion of tri- and tetra-sialylated glycans than follitropin alfa and also has both α2,3- and α2,6-linked sialic acid whereas follitropin alfa only has α2,3-linked sialic acid (72). *In vitro*, follitropin delta was observed to be equivalent to follitropin alfa in a cell-free FSH-receptor binding assay and in transfected Human Embryonic Kidney (HEK) 293 cells and cultured human granulosa cells (73). The differing pharmacokinetic (PK) and pharmacodynamic (PD) profiles of follitropin alfa and follitropin delta are likely to contribute to the observed differences in the properties of the two products in women, as well as to influence their efficacy for the treatment of infertility (71). In contrast with follitropin alfa, the bioactivity of follitropin delta determined by the Steelman-Pohley bioassay, which uses an international reference standard of CHO-produced r-hFSH, does not directly predict the PD activity (71). This has been attributed to more rapid clearance of follitropin delta compared with follitropin alfa in rats, resulting in lower apparent potency (73). This means that follitropin delta cannot be dosed according to bioactivity or specific bioactivity, as other follitropins are, and is instead dosed by mass (μg). Additionally, the pharmacological differences between follitropin delta and follitropin alfa suggest that these agents cannot be directly substituted in clinical practice.

The risk/benefit balance of follitropin delta was considered positive by the regulatory agencies and the most frequent adverse reactions reported during clinical trials were headache, pelvic discomfort, OHSS, pelvic pain, nausea, adnexa uteri pain and fatigue (51). In healthy female volunteers, follitropin delta demonstrated higher exposure and lower serum clearance compared with follitropin alfa (72). A phase 3 study (ESTHER-1) compared individualized doses of follitropin delta (fixed-dose throughout treatment; start dose individualized based on BMI and body weight) with follitropin alfa (starting dose of 150 IU, with potential for subsequent adjustment, with a maximum allowed daily dose of 450 IU) for ovarian stimulation in 1,326 women. The starting dose of follitropin delta was 12 μg in patients with AMH <15 pmol/L and 0.10–0.19 μg/kg (maximum daily dose: 12 μg) in patients with AMH ≥15 pmol/L. This study demonstrated non-inferiority of follitropin delta to follitropin alfa for the co-primary endpoints of ongoing pregnancy rate (30.7 and 31.6%, respectively; difference −0.9% [95% confidence interval (CI) −5.9, 4.1%]) and ongoing implantation rate (35.2% and 35.8%, respectively; difference −0.6% [95% CI −6.1, 4.8%]), with fewer women treated with follitropin delta requiring OHSS preventative measures (74). The live birth rate was also similar with follitropin alfa and follitropin delta (29.8 and 30.7%, respectively; difference −0.9% [95% CI −5.8, 4.0%]). However, the initial follitropin alfa dose allowed in this study (150 IU) was at the lower end of the recommended range in the SmPC for women undergoing multifollicular development prior to ART (150–225 IU daily) (42) and this starting dose could not be individualized, whereas the dose in the follitropin delta arm was individualized according to clinical markers which reduces the comparability of outcomes (75). The EMA assessment report states that, in the ESTHER-1 trial, the non-inferiority of follitropin delta compared with follitropin alfa for ongoing pregnancy can be explained by the heterogeneity of responses in different age groups; non-inferiority was driven by the 15% of the study population aged ≥38 years (75), with non-inferiority not demonstrated for women aged ≤37. It has also been noted that there were a greater number of canceled cycles for poor response in the follitropin delta arm (76).

### Follitropin Epsilon

Follitropin epsilon (FSH-GEX; Glycotope, Germany) is a recombinant FSH produced using a human blood cell line derived from a myeloid leukemia cell line and is currently not marketed (77). The cell lines used result in a high degree of bisecting N-acetlyglucosamine, a high antennarity and a high degree of sialylation, in particular after enrichment of the acidic isoforms (78). In addition, follitropin epsilon is highly fucosylated and has a ratio of 2,3 to 2,6 sialylation of about 1:1 (78). This is different from follitropin alfa and follitropin beta, which do not have any bisecting N-acetylgalactosamines or 2,6 sialylation. In phase 1 studies, follitropin epsilon and follitropin alfa had similar PK (Table 1), whereas PD activity (follicle growth and serum inhibin B levels) was increased with follitropin epsilon compared with follitropin alfa (77). No Phase III studies have been registered in publicly-available clinical trial repositories for this product.

### Corifollitropin Alfa

Due to its short half-life FSH has to be injected daily, which may be inconvenient and an unacceptable burden to patients; longer-acting r-hFSH preparations are, therefore, being investigated (79). The only approved longer acting r-hFSH (FSH-CTP, corifollitropin alfa, Elonva; Merck Sharp Dohme, Kenilworth, NJ, USA) was developed via addition of the carbonyl-terminal peptide (CTP) of the β-subunit of hCG to the β-subunit of FSH, generating a chimeric protein. This prolonged the half-life of the r-hFSH without impacting on assembly with the α-subunit, or the secretion or action of the dimer (50, 80). Corifollitropin alfa received marketing approval in the EU in 2010 for use in women undergoing fertility treatment, it is currently not approved for use in men or in the USA (41). The risk/benefit balance of corifollitropin alfa is considered positive, with the most frequently reported adverse reactions during clinical trials being pelvic discomfort, OHSS, headache, pelvic pain, nausea, fatigue and breast tenderness. Corifollitropin alfa can be administered as a single subcutaneous injection to replace the first 7 days of daily FSH therapy, simplifying treatment, as it has a 2-fold longer half-life and almost four-fold longer time to peak serum level than other available FSH preparations (Table 1) (81, 82). Meta-analyses of RCTs comparing corifollitropin alfa and daily injections of r-hFSH in women receiving ART treatment found no significant differences in LBR, ongoing pregnancy rate (OPR) or clinical pregnancy rate (CPR) between the treatments (79, 83, 84). There was evidence of reduced LBR (co-primary endpoint) in women receiving a low dose (60 to 120 μg) of long-acting FSH compared with daily FSH (79). There was no significant increase in OHSS, however, a higher number of oocytes were stimulated with corifollitropin alfa than with r-hFSH, and there was higher cycle cancellation due to overstimulation with corifollitropin alfa (79, 83, 84). Further research is needed to determine whether long-acting FSH is safe and effective for use in hyper-responders and poor ovarian responders and in women with all causes of subfertility (79).

Other methods for prolonging the half-life of FSH have been attempted. These include increasing elimination time by adding the Fc domain of IgG to the FSH molecule (85, 86), addition of new glycosylation sites and N-terminal extensions, which result in larger molecules with increased charge (87) and tethering two copies of the N-linked glycosylation signal sequence between the α- and β-subunits of hFSH, creating a single-chain fusion hormone analog (88).

### Differences Between Recombinant and Urinary Follicle-Stimulating Hormone Preparations

Two systematic reviews have compared r-hFSH (any preparation) with urinary gonadotropins (89, 90). The first compared r-hFSH with urinary gonadotropins (hMG, purified urinary FSH [u-FSH] or highly-purified u-FSH) in women undergoing ART, and included 42 trials (9,606 patients); there was no significant difference in LBR (28 trials [7,339 patients]; odds ratio [OR] 0.97, 95% CI 0.87, 1.08) or OHSS incidence (32 trials [7,740 patients]; OR 1.18, 95% CI 0.86, 1.61) between the two types of FSH preparation (89). When only fresh cycles were considered the difference in LBR (25 trials [4,952 patients]; odds ratio [OR] 0.97, 95% CI 0.85, 1.11) remained (89). Similarly, in a comparison of r-hFSH with urinary gonadotropins (hMG or u-FSH) for ovulation induction in patients with polycystic ovary syndrome (PCOS), there was no difference in LBR (co-primary endpoint; five trials [505 patients]; OR 1.21, 95% CI 0.83, 1.78) or CPR (secondary endpoint; eight trials [1,330 patients]; OR 1.05, 95% CI 0.88, 1.27) with the two FSH preparations (90). There was also no difference in the incidence of OHSS (co-primary endpoint) between r-hFSH and u-FSH (10 trials [1,565 patients]; OR 1.52, 95% CI 0.81, 2.84) or between r-hFSH and hMG (two trials [52 patients]; OR 9.95, 95% CI 0.47, 210.19) (90). Although authors of both reviews concluded that there was likely to be little if any clinical difference between r-hFSH and urinary gonadotropins (89, 90), authors of the latter review considered the available evidence to be of low or very low quality (90).

These results are in general agreement with those of a meta-analysis that compared r-hFSH with highly-purified hMG in ART using data from a total of 16 studies (4,040 patients) (91). When adjusted for baseline conditions, hMG treatment was associated with fewer oocytes (primary endpoint; −2.10, 95% CI −2.83, −1.36) and a higher required dose (secondary endpoint; mean difference 235 IU, 95% CI: 16.62, 454.30) but a similar pregnancy rate (secondary endpoint; risk ratio [RR] 1.10, 95% CI 0.97, 1.25) (91).

These meta-analyses predominantly included fresh cycles, for example, the meta-analysis by van Wely et al. only included three trials that studied frozen-thawed embryo transfer in addition to fresh embryo transfer (89). This is because, for many years, IVF success was measured per fresh cycle or embryo transfer. As freezing and thawing technology has improved, this definition has been challenged and it has been suggested that IVF success should instead be evaluated as cumulative live birth rates (CLBR), defined as the first live birth following the use of all fresh and frozen embryos derived from a single ovarian stimulation cycle (92, 93). A positive correlation has been observed between live birth rate per cycle and number of oocytes retrieved, up to 15 oocytes (*p* < 0.001 for comparison between age groups) (94–97). When the association between CLBR and number of oocytes was evaluated, the association remained (98–100). For example, in an analysis by Polyzos et al. the OR (95% CI) for live birth in the second and third cycle was 1.18 (1.07–1.30) for women with 4–9 aspirated oocytes in the first cycle, 1.41 (1.27–1.57) for women with 10–15 aspirated oocytes and 1.63 (1.42–1.88) for women with more than 15 aspirated oocytes compared with patients with 0–3 aspirated oocytes. In several studies a greater number of oocytes were retrieved when r-hFSH rather than urinary gonadotropins were used (101). This suggests that owing to the higher number of oocytes retrieved with r-hFSH compared with urinary gonadotropins, CLBR might be higher when r-hFSH is used.

In clinical trials comparing originator follitropin alfa (GONAL-f) with highly-purified u-FSH (Metrodin HP) in women undergoing ART, the mean number of oocytes obtained with r-hFSH was significantly higher than that obtained with u-FSH (102, 103). There was no difference in CPR (secondary endpoint; 45 and 48%, respectively) (102) or LBR (secondary endpoint; 36 and 36%, respectively) (103), but singleton pregnancies were more common with u-FSH (102, 103). When follitropin beta (Puregon) and highly-purified u-FSH (Metrodin HP) were compared in women undergoing IVF, the mean number of oocytes (primary endpoint; 9.7 vs. 8.9; 95% CI for the difference: −1.7, 3.2) and CPRs did not differ significantly between treatment groups (secondary endpoints; per attempt, 35.4 vs. 26.6%, respectively [95% CI for the difference: −12.1, 29.6]; per transfer, 40.8 vs. 28.6%, respectively [95% CI for the difference: −10.3, 34.8]) (104).

### r-hFSH for the Treatment of Male Infertility

FSH plays an important role in spermatogenesis, stimulating the Sertoli cells to facilitate germ cell differentiation. Follitropin alfa and follitropin beta are approved for clinical use in males who have congenital or acquired hypogonadotropic hypogonadism, for the stimulation of spermatogenesis with concomitant hCG therapy (42, 46). In a small study (*N* = 8), r-hFSH (follitropin alfa) was observed to induce testicular growth, spermatogenesis and fertility, with acceptable tolerability, in men with gonadotropin deficiency; the magnitude of effect was considered to be similar to that achieved historically with u-FSH when used to restore normal fertility in men with gonadotropin deficiency (105). In a second larger study, 15 of 19 men treated with r-hFSH and hCG achieved spermatogenesis (106). A Cochrane review evaluating gonadotropins for idiopathic male factor subfertility, identified six RCTs including 456 patients, and observed a higher spontaneous pregnancy rate per couple with gonadotropin treatment compared with placebo/no treatment (five studies [412 patients]; OR 4.94, 95% CI 2.13, 11.44) (107). This review noted that reporting of adverse event data was sparse. However, the risk/benefit balance in males is considered positive (40).

### Human Chorionic Gonadotropin

Recombinant hCG (r-hCG) is produced in a CHO cell line in a similar manner to r-hFSH (55, 108) and is suitable for subcutaneous injection and self-administration (109). In healthy subjects, the PK (Table 2) and PD profiles of r-hCG are consistent with endogenous hCG physiology and similar to those seen with urinary hCG (u-hCG) (111). The elimination half-lives of r-hCG and u-hCG are comparable (29–30 h for r-hCG 250 μg vs. 35 h for u-hCG 5000 IU) as are the areas under the concentration-time curve; however, u-hCG tends to be distributed and eliminated slightly slower than r-hCG (111).

**Table 2:** Pharmacokinetics of a single dose of subcutaneous choriogonadotropin alfa (r-hCG; dose and population not reported) and lutropin alfa (r-hLH) 75 IU to 40,000 IU in female volunteers (109, 110).

**Mean value**	**r-hCG**	**r-hLH**
Bioavailability (%)	40	60
t_1/2β_ (h)	30	≈10–12
CL (L/h)	0.2^a^	2

a *Measured after intravenous administration*.

*CL, clearance, C_max_, maximum plasma concentration; h, hours; t_1/2β_, terminal elimination half-life; t_max_, time to C_max_*.

In the late 1990s, Duffy et al. observed that r-hCG and u-hCG were equally effective for stimulating steroidogenic and peptidergic activities of the corpus luteum during simulated early pregnancy in rhesus monkeys (112). The equipotency of r-hCG and u-hCG was also demonstrated in macaque monkeys, with the numbers of oocytes resuming meiosis and undergoing IVF being similar in animals treated with either the recombinant or urinary product (113). However, the bioactivity of r-hCG was greater than that of u-hCG, when administered at the same dose (measured in IU), as determined by a mouse Leydig cell bioassay validated for macaque serum (*p* < 0.05) (113). Subsequently, in 2001, r-hCG (choriogonadotropin alfa, Ovitrelle; Merck KGaA, Darmstadt, Germany) was licensed for clinical use as a trigger for final follicular maturation/ovulation and luteinisation after stimulation of follicular growth (109).

Three randomized, double-blind, double-dummy, parallel-group, multicentre trials have confirmed the similar efficacy of r-hCG and u-hCG. In one, there were no observed differences following treatment with r-hCG or u-hCG in the number of oocytes retrieved (primary endpoint; mean ± standard deviation [SD] 10.8 ± 4.5 vs. 10.3 ± 5.1) or the number of patients pregnant (secondary endpoint; 10 in each group) and adverse events were generally mild or moderate among the 84 women undergoing IVF or intracytoplasmic sperm injection (ICSI) and embryo transfer (114). Similarly, a multinational study in anovulatory or oligo-ovulatory patients showed that r-hCG administration resulted in the same rates of ovulation and pregnancy as u-hCG administration (115). Overall, 162 of the 177 patients (91.5%) in the per protocol population ovulated (primary endpoint): 95.3% receiving r-hCG and 88.0% receiving u-hCG; however, in this study, r-hCG was better tolerated than u-hCG (115). The European Recombinant Human Chorionic Gonadotropin Study Group compared the efficacy and safety of r-hCG and u-hCG for inducing final follicular maturation and early luteinisation in 172 evaluable women undergoing ovulation induction for ART (116). The primary endpoint, the mean number of oocytes retrieved per patient was not significant between treatments (11.6 for r-HCG and 10.6 for u-hCG; two-sided 90% CI for the difference: −0.841, 1.515). Patients treated with r-hCG demonstrated better outcomes for number of mature oocytes (9.4 and 7.1 with r-hCG and u-hCG, respectively; *p* = 0.027), serum progesterone (day 1 post hCG administration: 30.1 vs. 23.3 nmol/L [*p* = 0.04]; day 6–7 post hCG administration: 391.9 vs. 315.9 nmol/L [*p* = 0.03]) and hCG (day of embryo transfer: 2.1 μg/L vs. 1.6 μg/L [*p* = 0.0001]) levels, CPR (32 [33.0%] and 23 [24.7%] with r-hCG, and u-hCG, respectively), and LBR (26 [26.8%] and 21 [22.6%] with r-hCG and u-hCG, respectively). While both treatments were well tolerated, the incidence of adverse events was significantly higher in patients treated with u-hCG. Injection site reactions being the most common adverse events in with both treatments in these latter two studies (115, 116). Investigators concluded, that for triggering ovulation, r-hCG may have significant advantages over u-hCG (116).

Treatment with r-hCG and u-hCG was also shown to result in similar numbers of oocytes (primary endpoint) and 2PN oocytes (secondary endpoint) obtained in a prospective, open, randomized study in 275 women requiring induction of final follicular maturation and luteinisation for IVF with embryo transfer (117). In this study, the tolerability of r-hCG and u-hCG was similar, with >95% of injections with either hCG producing no adverse reactions. More recently, Bellavia et al. reported that highly purified u-hCG was not inferior to r-hCG with regard to the mean number of oocytes retrieved (13.3 vs. 12.5), with no differences observed in fertilization rate (57.3% [467/815] vs. 61.3% [482/787]) or tolerability between the hCG preparations (118).

### Luteinizing Hormone

Recombinant human luteinizing hormone (r-hLH, lutropin alfa, Luveris; Merck KGaA, Darmstadt, Germany) received marketing authorization for clinical use in 2000 in Europe and 2004 in the US (subsequently withdrawn at Merck KGaA's request in 2016) (110). r-hLH is produced in a similar manner to FSH in CHO cells transfected with vectors encoding the α and β subunits (119), and is suitable for subcutaneous injection and self-administration (110). The PK of r-hLH is almost identical to the LH component of hMG (Pergonal; Laboratoires Serono, Aubonne, Switzerland) with a terminal half-life of ~10–12 h (Table 2) (120). It should be highlighted that at the time of this analysis the LH component of hMG preparations was predominantly the LH component of post-menopausal urine, rather than hCG as is more common in later and currently available more highly purified preparations. r-hLH is approved for use in women with severe LH and FSH deficiency, in combination with r-hFSH (121). In specific countries outside Europe (Russia, Mexico) r-hLH is also approved for patients with suboptimal ovarian response in the context of ART treatment (122). To improve convenience, a 2:1 fixed-ratio combination of r-hFSH and r-hLH has also been developed (Pergoveris; Merck KGaA, Darmstadt, Germany), which received marketing approval in Europe in 2007 (123). Pergoveris is not currently approved in the US.

In women with severe FSH and LH deficiency, r-hLH has been shown to support r-hFSH-induced follicular development (124, 125). In an open-label, dose-finding study, in which women with hypogonadotropic hypogonadism were randomized to receive r-hLH in combination with r-hFSH 150 IU, 0, 14.3, 66.7, and 88.0% of women treated with r-hLH 0 IU (*n* = 8), 25 IU (*n* = 7), 75 IU (*n* = 9) and 225 IU (*n* = 10), respectively, had good or excessive follicular growth (*p* < 0.01 by Cochran-Armitage trend test for difference between groups) (125). This study demonstrated that although LH requirements varied, a minimum effective daily dose of 75 IU provides adequate follicular development and steroidogenesis. A second study confirmed that r-hFSH 150 IU plus r-hLH 75 IU is the most appropriate dose schedule for hypogonadotropic anovulatory women, with sufficient follicular growth observed in 94% (79/84) of initiated cycles (five cycles in three patients required a dose increase) and pregnancy achieved by 15 of the 38 treated women (39.5%) (126). A study in 169 women aged 38–42 years randomized to receive a combination of r-hFSH:r-hLH in one of four ratios: 1:0, 1:1, 2:1, or 3:1 (127). The starting dose of r-hFSH was 225 IU, with r-hLH dosed according to the ratio, and the dose of r-hFSH could be adjusted up to 450 IU. A greater mean number of oocytes was retrieved in the group receiving 2:1 r-hFSH:r-hLH compared with those receiving 1:1 and 3:1 r-hFSH:r-hLH (8.4, 7.4, and 7.5, respectively), and the adjusted clinical pregnancy rate was higher in the groups receiving 3:1 or 2:1 r-hFSH:r-hLH (12.2 and 12.0%, respectively) compared with those receiving 1:0 and 1:1 r-hFSH:r-hLH (4.6 and 2.4%, respectively).The 2:1 fixed-ratio is supported by the dose-finding and confirmatory studies in women with hypogonadotropic hypogonadism, as well as the ART study summarized here.

The ESPART study was an RCT evaluating the effect of fixed-ratio (2:1) combination r-hFSH:r-hLH compared with r-hFSH alone for controlled ovarian stimulation in 939 women with POR (128, 129). In the ESPART study, to be defined as having POR, women had to meet at least two of the following criteria: advanced maternal age (≥40–<41 years); a previous ART cycle with ≤3 oocytes retrieved with a conventional stimulation protocol; an abnormal ovarian reserve test characterized by an AMH level between 0.12 and 1.3 ng/ml, inclusive. There were no differences observed in efficacy outcomes (number of oocytes retrieved [primary endpoint]; biochemical pregnancy rate, CPR, OPR; and LBR) between patients receiving r-hFSH/r-hLH and those receiving r-hFSH alone. However, a *post-hoc* analysis of the ESPART study observed a higher live birth rate with r-hLH supplementation in patients with moderate or severe POR, while a higher live birth rate was observed with r-hFSH alone in patients with mild POR (130).

Five recent meta-analyses have evaluated whether supplementation of FSH with LH for controlled ovarian stimulation (COS) might improve ART outcomes (131–135). While LBR is the preferred outcome, reality has shown that LBR is only reported in a small proportion of available studies, and most papers report intermediate pregnancy outcomes (such as CPR or OPR), representing relevant outcomes to measure clinical treatments benefits in reproductive medicine when pregnancy losses are not impacted (136–139). These meta-analyses have reported some conflicting results, despite there being overlap among the studies included.

These meta-analyses have relied on RCTs conducted in the general population, and either suggest that there is no beneficial effect from LH supplementation or that LH supplementation to FSH results in improvements in some outcomes in these patients. A higher number of oocytes were retrieved without LH supplementation (primary endpoint; 29 studies [5,840 patients] standard mean difference −0.20, 95% CI −0.38, −0.02; *p* = 0.03) in one meta-analysis (135), whereas no difference in this endpoint was observed in another meta-analysis (primary endpoint; 43 studies [6,341 patients]; RR 1.17, 95% CI 0.42, 1.92; *p* = 0.002) (133). A higher pregnancy rate (secondary endpoint; 29 studies [5,565 patients] OR 1.20, 95% CI 1.06, 1.37) was observed by one meta-analysis (135), whereas in other meta-analyses a higher CPR (secondary endpoint; 43 studies [6,393 patients]; RR 1.3, 95% CI 1.05, 1.62; *p* = 0.016) (133), higher OPR (secondary endpoint: 19 studies [3,129 patients] OR 1.20, 95% CI 1.01, 1.42) (134) and higher LBR (primary endpoint: 4 studies [499 patients] OR 1.32, 95% CI 0.85, 2.06; secondary endpoint: 39 studies [6,237 patients] RR 1.11, 95% CI 1.01, 1.21) (133, 134) were observed with LH supplementation to FSH compared with FSH alone. These findings may reflect the different characteristics of the pooled populations, depending on the trials included.

It has been suggested that the benefits of LH supplementation may occur in subpopulations characterized by LH insufficiency, including hypo–responders (133, 134). Hypo-response is characterized by an unexpected resistance to ovarian stimulation with standard doses of gonadotropins. This resistance might be diagnosed in women with otherwise normal ovarian reserve during ovarian stimulation who demonstrate an initial slow response and observed through serum estradiol levels and follicular growth or diagnosed retrospectively where higher-than-expected gonadotropin doses have been used (140). In patients with poor ovarian response (POR; including hypo-responders), supplementation with LH results in increased CPR (*post-hoc* analysis: RR 1.3, 95% CI 1.05, 1.62; p = 0.016) (133), OPR (subgroup analysis: 3 trials [79 patients] OR 2.06, 95% CI 1.20, 3.53) (134) and LBR (*post-hoc* analysis: RR 1.30, 95% CI 0.95, 1.78) (133).

When only hypo-responders were considered, supplementation with LH did not increase the number of oocytes retrieved (two RCTs and one cohort study [319 patients] OR 1.98, 95% CI 0.17, 3.80; *p* = 0.03), but did increase implantation rate (four RCTS and one cohort study [766 patients] OR 2.62, 95% CI 1.37, 4.99; *p* = 0.004), and CPR (three RCTs and one cohort study [361 patients] OR 2.03, 95% CI 1.27, 3.25; *p* = 0.003) (132) compared with FSH alone. LBR could not be evaluated by this meta-analysis as it was only included as an endpoint in one study (132).

A systematic review (without meta-analysis) that assessed the effect of r-hLH supplementation in COS as part of ART in six different patient populations (prevention of OHSS; women with profoundly suppressed LH levels after administration of a gonadotropin-releasing hormone [GnRH] agonist; women co-treated with a GnRH antagonist; women with a hypo-response to r-hFSH; women of advanced reproductive age; and women with POR, including women meeting the ESHRE Bologna criteria) identified two populations that may benefit from this treatment approach (131). In women with a hypo-response to r-hFSH the evaluated literature suggests that a greater number of oocytes might be retrieved and a higher implantation rate obtained with LH plus FSH compared with FSH alone (based on two studies). In women of advance reproductive age a higher implantation rate may be obtained with LH plus FSH compared with FSH alone (based on four studies). A lower proportion of patients with OHSS were observed with LH supplementation in patients when used for prevention of OHSS. No difference between treatment with LH plus FSH and FSH alone was observed in women with profoundly suppressed LH levels after administration of a GnRH agonist, women co-treated with a GnRH antagonist and poor ovarian responders.

## Oral Gonadotropins

All gonadotropin preparations have to be injected, which increases the treatment burden for patients. There has therefore been interest in producing a product that can be dosed orally. It is not possible to dose gonadotropins orally because they are proteins and will not be absorbed, rather they are digested by enzymes. As a result of this, attempts to produce an oral drug for ovarian stimulation have focussed on FSH agonists. One oral FSH agonist has been evaluated in healthy females but no effect on follicular development was observed, which was eventually attributed to the low doses used (141). Non-conclusive data is available for this option nowadays.

## Injection Devices

Animal-derived and urinary gonadotropin products had to be injected intramuscularly using a syringe and vial, with reconstitution required before injection. Owing to the increased purity of recombinant products, a smaller injection volume is required and these can be injected subcutaneously using smaller gauge needles. In addition, these products have greater stability and liquid formulations of recombinant products have been produced, removing the need for reconstitution before injection. This in turn has enabled the development of pen injection devices, which are designed to improve ease-of-use and patient convenience, including the ability to both select the starting dose with greater precision (in increments as low as 12.5 IU) and adapt the dose during treatment, based on treatment response in small increments (12.5 IU) (142–144).

## Conclusions

ART has come a long way since 1927, when gonadotropins were first identified, and currently available gonadotropin preparations better enable treatment individualization as part of patient-centered care. Patient-centeredness should be an aspect of all consultations and treatment decisions relating to medically assisted reproduction treatment. This should include discussions of whether treatment is appropriate, and if it is appropriate, which treatment would be most favorable. This treatment should be individualized according to the characteristics of the patient(s) and monitored to ensure that effectiveness is optimal, based on treatment response and safety, with treatment adjusted during treatment if it is not. The availability of recombinant products, which provide a pure form of the gonadotropin and can be accurately dosed, has improved the ability of medical practitioners to individualize treatment in this manner. Currently available products can be injected subcutaneously rather than intramuscularly, and pen injection devices are available, improving ease-of-use and more precise dose selection and adaption (in 12.5 IU dose increments). Work to develop new preparations is continuing, and a goal must remain the development of orally active FSH agonists and antagonists.

## Author Contributions

All authors listed have made a substantial, direct and intellectual contribution to the work, and approved it for publication.

### Conflict of Interest Statement

WB is an employee of Merck Serono GmbH, Darmstadt, Germany. SL and TD are employees of Merck Healthcare KGaA, Darmstadt, Germany. VA is an employee of EMD Serono, Rockland, MA, USA, a business of Merck KGaA, Darmstadt, Germany. SS has received grants and non-financial support from Merck and Ferring. The remaining author declares that the research was conducted in the absence of any commercial or financial relationships that could be construed as a potential conflict of interest.

## Abbreviations

AMH: anti-Müllerian hormone
ART: assisted reproductive technologies
BMI: body mass index
CHO: Chinese hamster ovary
CI: confidence interval
CLBR: cumulative live birth rate
COS: controlled ovarian stimulation
CPR: clinical pregnancy rate
CTP: carbonyl-terminal peptide
EMA: European Medicine Agency
ESHRE: European Society of Human Reproduction and Embryology
EU: European Union
Fc: Fragment crystallisable
FSH: follicle-stimulating hormone
GnRH: gonadotropin releasing hormone
hCG: human chorionic gonadotropin
HEK: human embryonic kidney
hMG: human menopausal gonadotropin
HPLC: high performance liquid chromatography
ICSH: interstitial cell stimulating hormone
ICSI: intracytoplasmic sperm injection
IgG: immunoglobulin G
IRP: international reference product
IU: international units
IVF: *in vitro* fertilization
LBR: live birth rate
LH: luteinizing hormone
OHSS: ovarian hyperstimulation syndrome
OPR: ongoing pregnancy rate
OR: odds ratio
PCOS: polycystic ovary syndrome
PD: pharmacodynamics
PK: pharmacokinetics
POR: poor ovarian response
RCT: randomized controlled trial
r-hCG: recombinant human chorionic gonadotropin
r-hFSH: recombinant human follicle-stimulating hormone
r-hLH: recombinant human luteinizing hormone
RR: risk ratio
u-FSH: urinary follicle-stimulating hormone
u-hCG: urinary human chorionic gonadotropin
USA: United States of America
WHO: World Health Organization.

## References

[1] Ascheim S Zondek B Hypophysenvorderlappenhormone und ovarialhormone im harn von schwangeren. Klin Wochenschr. (1927) 6:13 10.1007/BF01728562

[2] Lunenfeld B Ezcurra D D'Hooghe T The development and evolution of gonadotropins in ART. Fertil Steril. (2018) 110:255–62. 10.1016/j.fertnstert.2018.06.005

[3] Seegar-Jones GE Gey GO Ghisletta M Hormone production by placental cells maintained in continuous culture. Bull Johns Hopkins Hosp. (1943) 72:26–38.

[4] Lunenfeld B Gonadotropin stimulation: past, present and future. Reprod Med Biol. (2012) 11:11–25. 10.1007/s12522-011-0097-2 29699102 PMC5906949

[5] Smith PE Engle ET Experimental evidence of the role of anterior pituitary in development and regulation of gonads. Am J Anat. (1927) 40:159 10.1002/aja.1000400202

[6] Zondek B Weitere untersuchungen zur darstellung, biologie und klinik des hypophysenvorderlappenhormones (Prolan). Zentralblatt fur Gynakologie. (1929) 14:834–48.

[7] Lunenfeld B. Historical perspectives in gonadotrophin therapy. Hum Reprod Update. (2004) 10:453–67. 10.1093/humupd/dmh044 15388674

[8] D'Amour FE D'Amour MC The biologic potency of international standrad chorionic gonadotropin. Endocrinology. (1940) 26:93–6. 10.1210/endo-26-1-93

[9] Gurin S Bachman G Wilson DW The gonadotropic hormone of urine of pregnancy. ii) Chemical studies of preparations having high biological activity. J Biol Chem. (1940) 133:467.

[10] Katzman PA Godfried M Cain CK Doisy EA The preparation of chorionic gonadotrophin by chromatographic adsorption. J Chem Biol. (1943) 148:501–7.

[11] Greep RO Van Dyke HB Chan BF Gonadotropins of the swine pituitary: various biological effects of purified thylakentrin (FSH) and pure metakentrin (ICSH). Endocrinolology. (1942) 30:635–49. 10.1210/endo-30-5-635

[12] Steelman SL Lamont WA Baltes BJ. Preparation of highly active follicle stimulating hormone from swine pituitaries. Endocrinology. (1955) 56:216–7. 10.1210/endo-56-2-216 13231832

[13] Cole HH Hart GH The potency of blood serum of mares in progressive stages of pregnancy in affecting the sexual maturity of the immature rat. Am J Physiol. (1930) 93:57 10.1152/ajplegacy.1930.93.1.57

[14] Mazer C Ravetz E The effect of combined administration of chorionic gonadotropin and the pituitary synergist on the human ovary. Am J Obstet Gynaecol. (1941) 41:474–588. 10.1016/S0002-9378(41)90825-6

[15] Leathem JH Rakoff A. Gonadotrophic hormone therapy in man complicated by antihormone formation. Am J Obstet Gynecol. (1948) 56:521–6. 10.1016/0002-9378(48)90638-3 18877183

[16] Gemzell C. Human pituitary gonadotropins in the treatment of sterility. Fertil Steril. (1966) 17:149–59. 10.1016/S0015-0282(16)35880-0 5907039

[17] Gemzell CA Diczfalusy E Tillinger G. Clinical effect of human pituitary follicle-stimulating hormone (FSH). J Clin Endocrinol Metab. (1958) 18:1333–48. 10.1210/jcem-18-12-1333 13611018

[18] Cochius JI Burns RJ Blumbergs PC Mack K Alderman CP. Creutzfeldt-Jakob disease in a recipient of human pituitary-derived gonadotrophin. Aust N Z J Med. (1990) 20:592–3. 10.1111/j.1445-5994.1990.tb01322.x 2222355

[19] Cochius JI Hyman N Esiri MM. Creutzfeldt-Jakob disease in a recipient of human pituitary-derived gonadotrophin: a second case. J Neurol Neurosurg Psychiatry. (1992) 55:1094–5. 10.1136/jnnp.55.11.1094 1469410 PMC1015303

[20] Dumble LJ Klein RD. Creutzfeldt-Jakob legacy for Australian women treated with human pituitary gonadotropins. Lancet. (1992) 340:847–8. 10.1016/0140-6736(92)92720-Z 1357261

[21] Borth R Lunenfeld B de Watteville H Active gonadotrope d'un extrait d'urines de femmes en menopause. Experientia. (1954) 10:266–8. 10.1007/BF02157401 13183071

[22] Albert A Human Pituitary Gonadotropins- a workshop conference. In: Charles C Thomas December 1959. Gatlinburg, TN: Springfield (1961).

[23] Borth R Lunenfeld B De Watteville H. Day-to-day variation in urinary gonadotrophin and steroid levels during the normal menstrual cycle. Fertil Steril. (1957) 8:233–54. 10.1016/S0015-0282(16)61356-0 13427790

[24] Lunenfeld B Menzi A Volet B. Clinical effects of human postmenopausal gonadotropin. In: Fuchs F editor. Advance Abstracts of Short Communications, 1st International Congress of Endocrinology. Copenhagen: Periodical (1960).

[25] Lunenfeld B Rabau E Rumney G Winkelsberg G The responsiveness of the human ovary to gonadotropin (Hypophysis III). Proc Third World Cong Gynecol Obstet. (1961) 1:22.

[26] Lunenfeld B Sukmovici S Rabau E Eshkol A L'induction de l'ovulation dans les amenorrees hypophysaires par un traitement combini de gonadotrophines unnaires minopausiques et de gonadotrophines chononiques. CR Soc FT Gynecol. (1962) 5:1–6.

[27] Lunenfeld B Treatment of anovulation by human gonadotropins. J Int Fed Gynecol Obstet. (1963) 1:15 10.1002/j.1879-3479.1963.tb00335.x

[28] World Health Organization Expert Committee on Biological Standardization (Chair B.Lunenfeld), Vol 565. Technical Report Series. Geneva: World Health Organization (1972).

[29] Steelman SL Pohley FM. Assay of the follicle stimulating hormone based on the augmentation with human chorionic gonadotropin. Endocrinology. (1953) 53:604–16. 10.1210/endo-53-6-604 13116950

[30] WHO Expert Committee. *Agents Stimulating Gonadal Function in Human* (Chair B. Lunenfeld). Technical Report Series. WHO (1973). 4632594

[31] Steptoe PC Edwards RG. Birth after the reimplantation of a human embryo. Lancet. (1978) 2:366. 10.1016/S0140-6736(78)92957-4 79723

[32] Inge GB Brinsden PR Elder KT. Oocyte number per live birth in IVF: were steptoe and edwards less wasteful? Hum Reprod. (2005) 20:588–92. 10.1093/humrep/deh655 15689347

[33] Jones HW Jr. The use of controlled ovarian hyperstimulation (COH) in clinical *in vitro* fertilization: the role of georgeanna seegar jones. Fertil Steril. (2008) 90:e1–3. 10.1016/j.fertnstert.2007.07.1333 17905235

[34] Jones HW Jr Jones GS Andrews MC Acosta A Bundren C Garcia J. The program for *in vitro* fertilization at Norfolk. Fertil steril. (1982) 38:14–21. 10.1016/S0015-0282(16)46390-9 7095165

[35] Donini P Puzzuoli D D'Alessio I Lunenfeld B Eshkol A Parlow AF. Purification and separation of follicle stimulating hormone (FSH) and luteinizing hormone (LH) from human postmenopausal gonadotrophin (HMG). II. Preparation of biological apparently pure FSH by selective binding of the LH with an anti-HGG serum and subsequent chromatography. Acta Endocrinol. (1966) 52:186–98. 10.1530/acta.0.0520186 5952830

[36] Ferring Pharmaceuticals Ltd Menopur 75IU. (2015). Available online at: https://www.medicines.org.uk/emc/medicine/4322 (accessed March 28, 2019).

[37] Howles C Tanaka T Matsuda T. Management of male hypogonadotrophic hypogonadism. Endocr J. (2007) 54:177–90. 10.1507/endocrj.02-KR-98 17287584

[38] Lunenfeld B Mor A Mani M. Treatment of male infertility. I. human gonadotropins. Fertil Steril. (1967) 18:581–92. 10.1016/S0015-0282(16)36421-4 6037447

[39] Macleod J Pazianos A Ray BS. Restoration of human spermatogenesis by menopausal gonadotrophins. Lancet. (1964) 1:1196–7. 10.1016/S0140-6736(64)91212-7 14132660

[40] European Medicines Agency GONAL-f. (2018). Available online at: https://www.ema.europa.eu/en/medicines/human/EPAR/gonal-f (accessed January 29, 2019).

[41] Merck Sharp Dohme Limited Elonva. (2019) Available online at: https://www.ema.europa.eu/en/medicines/human/EPAR/elonva (accessed March 26, 2019).

[42] EMD Serono I Gonal-F. (2018). Available online at: https://www.drugs.com/pro/gonal-f.html (accessed 2019 26 Mar 2019).

[43] European Medicines Agency Ovaleap. (2018). Available online at: https://www.ema.europa.eu/en/medicines/human/EPAR/ovaleap (accessed January 29, 2019).

[44] European Medicines Agency Bemfola. (2018). Available online at: https://www.ema.europa.eu/en/medicines/human/EPAR/bemfola (accessed January 29, 2019).

[45] Drugs.com. Follistim AQ Approval History. (2018). Available online at: https://www.drugs.com/history/follistim-aq.html (accessed March 26, 2019).

[46] European Medicines Agency Puregon. (2018). Available online at: https://www.ema.europa.eu/en/medicines/human/EPAR/puregon (accessed January 29, 2019).

[47] Howles CM. Genetic engineering of human FSH (Gonal-F). Hum Reprod Update. (1996) 2:172–91. 10.1093/humupd/2.2.172 9079412

[48] Olijve W de Boer W Mulders JW van Wezenbeek PM. Molecular biology and biochemistry of human recombinant follicle stimulating hormone (Puregon). Mol Hum Reprod. (1996) 2:371–82. 10.1093/molehr/2.5.371 9238705

[49] Goa KL Wagstaff AJ. Follitropin alpha in infertility: a review. BioDrugs. (1998) 9:235–60. 10.2165/00063030-199809030-00006 18020563

[50] European Medicines Agency Elonva: Corifollitropin Alfa. (2015). Available online at: https://www.ema.europa.eu/en/medicines/human/EPAR/elonva (accessed March 10, 2019).

[51] European Medicines Agency Rekovelle: Follitropin Delta. (2017). Available online at: http://www.ema.europa.eu/ema/index.jsp?curl=pages/medicines/human/medicines/003994/human_med_002044.jsp&mid=WC0b01ac058001d124 (accessed June 21, 2018).

[52] le Contonnec JY Porchet HC Beltrami V Khan A Toon S Rowland M. Clinical pharmacology of recombinant human follicle-stimulating hormone. II. single doses and steady state pharmacokinetics. Fertil Steril. (1994) 61:679–86. 10.1016/S0015-0282(16)56645-X 8150110

[53] Voortman G Mannaerts BM Huisman JA. A dose proportionality study of subcutaneously and intramuscularly administered recombinant human follicle-stimulating hormone (Follistim^*^/Puregon) in healthy female volunteers. Fertil Steril. (2000) 73:1187–93. 10.1016/S0015-0282(00)00542-2 10856481

[54] de Leeuw R Mulders J Voortman G Rombout F Damm J Kloosterboer L. Structure-function relationship of recombinant follicle stimulating hormone (Puregon). Mol Hum Reproduct. (1996) 2:361–9. 10.1093/molehr/2.5.361 9238704

[55] Leao Rde B Esteves SC. Gonadotropin therapy in assisted reproduction: an evolutionary perspective from biologics to biotech. Clinics. (2014) 69:279–93. 10.6061/clinics/2014(04)10 24714837 PMC3971356

[56] Bassett RM Driebergen R. Continued improvements in the quality and consistency of follitropin alfa, recombinant human FSH. Reprod Biomed Online. (2005) 10:169–77. 10.1016/S1472-6483(10)60937-6 15823219

[57] Hugues J-N Durnerin IC. Gonadotrophins – filled-by-mass versus filled-by-bioassay. Reprod Biomed Online. (2005) 10:11–7. 10.1016/S1472-6483(11)60385-4 23577410

[58] Brinsden P Akagbosu F Gibbons LM Lancaster S Gourdon D Engrand P. A comparison of the efficacy and tolerability of two recombinant human follicle-stimulating hormone preparations in patients undergoing *in vitro* fertilization-embryo transfer. Fertil Steril. (2000) 73:114–6. 10.1016/S0015-0282(99)00450-1 10632423

[59] Harlin J Csemiczky G Wramsby H Fried G. Recombinant follicle stimulating hormone in *in-vitro* fertilization treatment-clinical experience with follitropin alpha and follitropin beta. Hum Reprod. (2000) 15:239–44. 10.1093/humrep/15.2.239 10655291

[60] Tulppala M Aho M Tuuri T Vilska S Foudila T Hakala-Ala-Pietila T. Comparison of two recombinant follicle-stimulating hormone preparations in *in-vitro* fertilization: a randomized clinical study. Hum Reprod. (1999) 14:2709–15. 10.1093/humrep/14.11.2709 10548606

[61] Williams RS Vensel T Sistrom CL Kipersztok S Rhoton-Vlasak A Drury K. Pregnancy rates in varying age groups after *in vitro* fertilization: a comparison of follitropin alfa (Gonal F) and follitropin beta (Follistim). Am J Obstet Gynecol. (2003) 189:342–6. 10.1067/S0002-9378(03)00728-2 14520188

[62] Mastrangeli R Satwekar A Cutillo F Ciampolillo C Palinsky W Longobardi S. *In-vivo* biological activity and glycosylation analysis of a biosimilar recombinant human follicle-stimulating hormone product (Bemfola) compared with its reference medicinal product (GONAL-f). PLoS ONE. (2017) 12:e0184139. 10.1371/journal.pone.0184139 28880909 PMC5589168

[63] de Mora F Fauser BCJM. Biosimilars to recombinant human FSH medicines: comparable efficacy and safety to the original biologic. Reprod Biomed Online. (2017) 35:81–6. 10.1016/j.rbmo.2017.03.020 28462793

[64] Winstel R Wieland J Gertz B Mueller A Allgaier H. Manufacturing of recombinant human follicle-stimulating hormone ovaleap((R)) (XM17), comparability with gonal-f((R)), and performance/consistency. Drugs R D. (2017) 17:305–12. 10.1007/s40268-017-0182-z 28386738 PMC5427053

[65] Orvieto R Seifer DB Biosimilar FSH preparations- are they identical twins or just siblings? Reprod Biol Endocrinol. (2016) 14:32 10.1186/s12958-016-0167-8 27301324 PMC4908720

[66] European Medicines Agency. Biosimilars in the EU. (2017). Available online at: https://www.ema.europa.eu/documents/leaflet/biosimilars-eu-information-guide-healthcare-professionals_en.pdf (accessed January 30, 2019).

[67] Rettenbacher M Andersen AN Garcia-Velasco JA Sator M Barri P Lindenberg S. A multi-centre phase 3 study comparing efficacy and safety of Bemfola((R)) versus Gonal-f((R)) in women undergoing ovarian stimulation for IVF. Reprod Biomed Online. (2015) 30:504–13. 10.1016/j.rbmo.2015.01.005 25735918

[68] Strowitzki T Kuczynski W Mueller A Bias P. Randomized, active-controlled, comparative phase 3 efficacy and safety equivalence trial of Ovaleap(R) (recombinant human follicle-stimulating hormone) in infertile women using assisted reproduction technology (ART). Reprod Biol Endocrinol. (2016) 14:1. 10.1186/s12958-015-0135-8 26733057 PMC4702416

[69] Papsch R Roeder C D'Hooghe T Longobardi S PMU40 - live birth rate (LBR), ongoing pregnancy rate (OPR) and ovarian hyperstimulation syndrome (OHSS) risk with originator versus biosimilar recombinant follitropin ALFA: a pooled analysis of clinical trial data. Value Health. (2018) 21:S314–S5. 10.1016/j.jval.2018.09.1876

[70] Strowitzki T Kuczynski W Mueller A Bias P. Safety and efficacy of Ovaleap(R) (recombinant human follicle-stimulating hormone) for up to 3 cycles in infertile women using assisted reproductive technology: a phase 3 open-label follow-up to Main Study. Reprod Biol Endocrinol. (2016) 14:31. 10.1186/s12958-016-0164-y 27287439 PMC4902897

[71] Koechling W Plaksin D Croston GE Jeppesen JV Macklon KT Andersen CY. Comparative pharmacology of a new recombinant FSH expressed by a human cell line. Endocrine Connect. (2017) 6:297–305. 10.1530/EC-17-0067 28450423 PMC5510450

[72] Olsson H Sandstrom R Grundemar L. Different pharmacokinetic and pharmacodynamic properties of recombinant follicle-stimulating hormone (rFSH) derived from a human cell line compared with rFSH from a non-human cell line. J Clin Pharmacol. (2014) 54:1299–307. 10.1002/jcph.328 24800998

[73] Therapeutic Goods Administration (TGA) Commonwealth of Australia Australian Public Assessment Report (AusPAR) Rekovelle. (2017). Available online at: https://www.tga.gov.au/sites/default/files/auspar-follitropin-delta-rhu-171025.docx (accessed March 29, 2019).

[74] Nyboe Andersen A Nelson SM Fauser BCJM García-Velasco JA Klein BM Arce J-C Individualized versus conventional ovarian stimulation for *in vitro* fertilization: a multicenter, randomized, controlled, assessor-blinded, phase 3 noninferiority trial. Fertil Steril. (2017) 107:387–96.e4. 10.1016/j.fertnstert.2016.10.033 27912901

[75] D'Hooghe T Longobardi S Letter to Editor in Response to: Individualized Versus Conventional Ovarian Stimulation for in vitro Fertilization: A Multicenter, Randomized, Controlled, Assessor-Blinded, Phase 3 Noninferiority Trial. (2017). Available online at: https://www.fertstertdialog.com/users/16110-fertility-and-sterility/posts/12852–23086 (accessed December 21, 2018).

[76] Wilkinson J Lensen S Letter to editor in response to: individualized versus conventional ovarian stimulation for *in vitro* fertilization: a multicenter, randomized, controlled, assessor-blinded, phase 3 noninferiority trial (2017) [cited 2019 26 Mar 2019]. Available online at: https://www.fertstertdialog.com/users/16110-fertility-and-sterility/posts/12852–23086.

[77] Abd-Elaziz K Duijkers I Stöckl L Dietrich B Klipping C Eckert K. A new fully human recombinant FSH (follitropin epsilon): two phase I randomized placebo and comparator-controlled pharmacokinetic and pharmacodynamic trials. Hum Reprod. (2017) 32:1639–47. 10.1093/humrep/dex220 28591833

[78] Glycotope GmbH inventor; Glycotope GmbH assignee. US Patent for Recombinant Human Follicle-Stimulating Hormone Patent. Patent # 9,527,899. US (2011).

[79] Pouwer AW Farquhar C Kremer JA Long-acting FSH versus daily FSH for women undergoing assisted reproduction. Cochrane Database Syst Rev. (2015) 2015:CD009577 10.1002/14651858.CD009577.pub3 PMC10415736 26171903

[80] Fares FA Suganuma N Nishimori K LaPolt PS Hsueh AJ Boime I. Design of a long-acting follitropin agonist by fusing the C-terminal sequence of the chorionic gonadotropin beta subunit to the follitropin beta subunit. Proc Natl Acad Sci USA. (1992) 89:4304–8. 10.1073/pnas.89.10.4304 1374895 PMC49070

[81] Fauser BCJM Alper MM Ledger W Schoolcraft WB Zandvliet A Mannaerts BMJL. Pharmacokinetics and follicular dynamics of corifollitropin alfa versus recombinant FSH during ovarian stimulation for IVF. Reprod Biomed Online. (2010) 21:593–601. 10.1016/j.rbmo.2010.06.032 20843746

[82] Ledger WL Fauser BCJM Devroey P Zandvliet AS Mannaerts BMJL. Corifollitropin alfa doses based on body weight: clinical overview of drug exposure and ovarian response. Reprod Biomed Online. (2011) 23:150–9. 10.1016/j.rbmo.2011.04.002 21665541

[83] Fensore S Di Marzio M Tiboni GM. Corifollitropin alfa compared to daily FSH in controlled ovarian stimulation for *in vitro* fertilization: a meta-analysis. J Ovarian Res. (2015) 8:33. 10.1186/s13048-015-0160-4 26036214 PMC4465305

[84] Mahmoud Youssef MA van Wely M Aboulfoutouh I El-Khyat W van der Veen F Al-Inany H. Is there a place for corifollitropin alfa in IVF/ICSI cycles? a systematic review and meta-analysis. Fertil Steril. (2012) 97:876–85. 10.1016/j.fertnstert.2012.01.092 22277766

[85] Low SC Nunes SL Bitonti AJ Dumont JA. Oral and pulmonary delivery of FSH–Fc fusion proteins via neonatal Fc receptor-mediated transcytosis. Hum Reprod. (2005) 20:1805–13. 10.1093/humrep/deh896 15817590

[86] Zhang Y-L Guo K-P Ji S-Y Liu X-M Wang P Wu J. Development and characterization of a novel long-acting recombinant follicle stimulating hormone agonist by fusing Fc to an FSH-β subunit. Hum Reprod. (2016) 31:169–82. 10.1093/humrep/dev295 26621853

[87] Perlman S van den Hazel B Christiansen J Gram-Nielsen S Jeppesen CB Andersen KV. Glycosylation of an N-terminal extension prolongs the half-life and increases the *in vivo* activity of follicle stimulating hormone. J Clin Endocrinol Metab. (2003) 88:3227–35. 10.1210/jc.2002-021201 12843169

[88] Klein J Lobel L Pollak S Lustbader B Ogden RT Sauer MV. Development and characterization of a long acting recombinant hFSH agonist. Hum Reprod. (2003) 18:50–6. 10.1093/humrep/deg024 12525440

[89] van Wely M Kwan I Burt AL Thomas J Vail A Van der Veen F Recombinant versus urinary gonadotrophin for ovarian stimulation in assisted reproductive technology cycles. Cochrane Database Syst Rev. (2011) 2011:CD005354 10.1002/14651858.CD005354.pub2 PMC7388278 21328276

[90] Weiss NS Kostova E Nahuis M Mol BWJ van der Veen F van Wely M. Gonadotrophins for ovulation induction in women with polycystic ovary syndrome. Cochrane Database Syst Rev. (2019) 1:CD010290. 10.1002/14651858.CD010290.pub3 30648738 PMC6353048

[91] Lehert P Schertz JC Ezcurra D. Recombinant human follicle-stimulating hormone produces more oocytes with a lower total dose per cycle in assisted reproductive technologies compared with highly purified human menopausal gonadotrophin: a meta-analysis. Reprod Biol Endocrinol. (2010) 8:112. 10.1186/1477-7827-8-112 20846363 PMC2954883

[92] Drakopoulos P Errazuriz J Santos-Ribeiro S Tournaye H Vaiarelli A Pluchino N Cumulative live birth rates in IVF. Minerva Ginecol. (2018) 360:236–243. 10.23736/S0026-4784.18.04347-2 30486636

[93] Maheshwari A McLernon D Bhattacharya S. Cumulative live birth rate: time for a consensus? Hum Reprod. (2015) 30:2703–7. 10.1093/humrep/dev263 26466912

[94] Baker VL Brown MB Luke B Conrad KP. Association of number of retrieved oocytes with live birth rate and birth weight: an analysis of 231,815 cycles of in vitro fertilization. Fertil Steril. (2015) 103:931–8 e2. 10.1016/j.fertnstert.2014.12.120 25638421 PMC4415984

[95] Briggs R Kovacs G MacLachlan V Motteram C Baker HW. Can you ever collect too many oocytes? Hum Reprod. (2015) 30:81–7. 10.1093/humrep/deu272 25362088

[96] Steward RG Lan L Shah AA Yeh JS Price TM Goldfarb JM. Oocyte number as a predictor for ovarian hyperstimulation syndrome and live birth: an analysis of 256,381 *in vitro* fertilization cycles. Fertil Steril. (2014) 101:967–73. 10.1016/j.fertnstert.2013.12.026 24462057

[97] Sunkara SK Rittenberg V Raine-Fenning N Bhattacharya S Zamora J Coomarasamy a. association between the number of eggs and live birth in IVF treatment: an analysis of 400 135 treatment cycles. Hum Reprod. (2011) 26:1768–74. 10.1093/humrep/der106 21558332

[98] Magnusson A Kallen K Thurin-Kjellberg A Bergh C. The number of oocytes retrieved during IVF: a balance between efficacy and safety. Hum Reprod. (2018) 33:58–64. 10.1093/humrep/dex334 29136154

[99] Malchau SS Henningsen AA Forman J Loft A Nyboe Andersen A Pinborg A. Cumulative live birth rate prognosis based on the number of aspirated oocytes in previous ART cycles. Hum Reprod. (2019) 34:171–80. 10.1093/humrep/dey341 30541039

[100] Polyzos NP Drakopoulos P Parra J Pellicer A Santos-Ribeiro S Tournaye H. Cumulative live birth rates according to the number of oocytes retrieved after the first ovarian stimulation for *in vitro* fertilization/intracytoplasmic sperm injection: a multicenter multinational analysis including approximately 15,000 women. Fertil Steril. (2018) 110:661–70 e1. 10.1016/j.fertnstert.2018.04.039 30196963

[101] Levi Setti PE Alviggi C Colombo GL Pisanelli C Ripellino C Longobardi S. Human recombinant follicle stimulating hormone (rFSH) compared to urinary human menopausal gonadotropin (HMG) for ovarian stimulation in assisted reproduction: a literature review and cost evaluation. J Endocrinol Invest. (2015) 38:497–503. 10.1007/s40618-014-0204-4 25480425 PMC4555088

[102] Bergh C Howles CM Borg K Hamberger L Josefsson B Nilsson L. Recombinant human follicle stimulating hormone (r-hFSH; Gonal-F) versus highly purified urinary FSH (Metrodin HP): results of a randomized comparative study in women undergoing assisted reproductive techniques. Hum Reprod. (1997) 12:2133–9. 10.1093/humrep/12.10.2133 9402268

[103] Frydman R Howles CM Truong F. A double-blind, randomized study to compare recombinant human follicle stimulating hormone (FSH; Gonal-F) with highly purified urinary FSH (Metrodin) HP) in women undergoing assisted reproductive techniques including intracytoplasmic sperm injection. The French Multicentre Trialists. Hum Reprod. (2000) 15:520–5. 10.1093/humrep/15.3.520 10686190

[104] Hedon B Out HJ Hugues JN Camier B Cohen J Lopes P. Efficacy and safety of recombinant follicle stimulating hormone (Puregon) in infertile women pituitary-suppressed with triptorelin undergoing *in-vitro* fertilization: a prospective, randomized, assessor-blind, multicentre trial. Hum Reprod. (1995) 10:3102–6. 10.1093/oxfordjournals.humrep.a135866 8822422

[105] Liu PY Turner L Rushford D McDonald J Baker HW Conway AJ. Efficacy and safety of recombinant human follicle stimulating hormone (Gonal-F) with urinary human chorionic gonadotrophin for induction of spermatogenesis and fertility in gonadotrophin-deficient men. Hum Reprod. (1999) 14:1540–5. 10.1093/humrep/14.6.1540 10357972

[106] Bouloux P Warne DW Loumaye E. Efficacy and safety of recombinant human follicle-stimulating hormone in men with isolated hypogonadotropic hypogonadism. Fertil Steril. (2002) 77:270–3. 10.1016/S0015-0282(01)02973-9 11821082

[107] Attia AM Abou-Setta AM Al-Inany HG Gonadotrophins for idiopathic male factor subfertility. Cochrane Database Syst Rev. (2013) 2013:CD005071 10.1002/14651858.CD005071.pub4 PMC11513186 23970458

[108] Youssef MA Abou-Setta AM Lam WS. Recombinant versus urinary human chorionic gonadotrophin for final oocyte maturation triggering in IVF and ICSI cycles. Cochrane Database Syst Rev. (2016) 4:CD003719. 10.1002/14651858.CD003719.pub4 27106604 PMC7133782

[109] European Medicines Agency Ovitrelle. (2018). Available online at: https://www.ema.europa.eu/en/medicines/human/EPAR/ovitrelle (accessed January 30, 2019).

[110] European Medicines Agency Luveris. (2018). Available online at: https://www.ema.europa.eu/en/medicines/human/EPAR/luveris (accessed January 30, 2019).

[111] Trinchard-Lugan I Khan A Porchet HC Munafo A. Pharmacokinetics and pharmacodynamics of recombinant human chorionic gonadotrophin in healthy male and female volunteers. Reprod Biomed Online. (2002) 4:106–15. 10.1016/S1472-6483(10)61927-X 12470572

[112] Duffy DM Hutchison JS Stewart DR Stouffer RL Stimulation of primate luteal function by recombinant human chorionic gonadotropin and modulation of steroid, but not relaxin, production by an inhibitor of 3 beta-hydroxysteroid dehydrogenase during simulated early pregnancy. J Clin Endocrinol Metab. (1996) 81:2307–13. 10.1210/jcem.81.6.8964869 8964869

[113] Zelinski-Wooten MB Hutchison JS Trinchard-Lugan I Hess DL Wolf DP Stouffer RL. Initiation of periovulatory events in gonadotrophin-stimulated macaques with varying doses of recombinant human chorionic gonadotrophin. Hum Reprod. (1997) 12:1877–85. 10.1093/humrep/12.9.1877 9363699

[114] Driscoll GL Tyler JP Hangan JT Fisher PR Birdsall MA Knight DC. A prospective, randomized, controlled, double-blind, double-dummy comparison of recombinant and urinary HCG for inducing oocyte maturation and follicular luteinization in ovarian stimulation. Hum Reprod. (2000) 15:1305–10. 10.1093/humrep/15.6.1305 10831560

[115] International Recombinant Human Chorionic Gonadotropin Study Group Induction of ovulation in World Health Organization group II anovulatory women undergoing follicular stimulation with recombinant human follicle-stimulating hormone: a comparison of recombinant human chorionic gonadotropin (rhCG) and urinary hCG. Fertil Steril. (2001) 75:1111–8. 10.1016/S0015-0282(01)01803-9 11384635

[116] European Recombinant Human Chorionic Gonadotrophin Study Group Induction of final follicular maturation and early luteinization in women undergoing ovulation induction for assisted reproduction treatment–recombinant HCG versus urinary HCG. the european recombinant human chorionic gonadotrophin study group. Hum Reprod. (2000) 15:1446–51. 10.1093/humrep/15.7.1446 10875887

[117] Chang P Kenley S Burns T Denton G Currie K DeVane G. Recombinant human chorionic gonadotropin (rhCG) in assisted reproductive technology: results of a clinical trial comparing two doses of rhCG (Ovidrel) to urinary hCG (Profasi) for induction of final follicular maturation in in vitro fertilization-embryo transfer. Fertil Steril. (2001) 76:67–74. 10.1016/S0015-0282(01)01851-9 11438321

[118] Bellavia M de Geyter C Streuli I Ibecheole V Birkhauser MH Cometti BP. Randomized controlled trial comparing highly purified (HP-hCG) and recombinant hCG (r-hCG) for triggering ovulation in ART. Gynecol Endocrinol. (2013) 29:93–7. 10.3109/09513590.2012.730577 23116325

[119] Shoham ZMD Insler VMD Recombinant technique and gonadotropins production: new era in reproductive medicine. Fertil Steril. (1998) 69:3S−15S. 10.1016/S0015-0282(97)00506-2 8690100

[120] le Cotonnec J-Y Porchet H Beltrami V Munafo A. Clinical pharmacology of recombinant human luteinizing hormone: part I. pharmacokinetics after intravenous administration to healthy female volunteers and comparison with urinary human luteinizing hormone. Fertil Steril. (1998) 69:189–94. 10.1016/S0015-0282(97)00501-3 9496327

[121] Merck Serono Luveris Summary of Product Characteristics. (2018). Available online at: https://www.medicines.org.uk/emc/product/1573/smpc (accessed July 24, 2019).

[122] Merck Pergoveris, Russian Summary of Product Characteristics. (2017).

[123] European Medicines Agency Pergoveris. (2018). Available online at: https://www.ema.europa.eu/en/medicines/human/EPAR/pergoveris (accessed January 30, 2019).

[124] Dhillon S Keating GM. Lutropin Alfa. Drugs. (2008) 68:1529–40. 10.2165/00003495-200868110-00005 18627209

[125] The European Recombinant Human LH Study Group Recombinant human luteinizing hormone (LH) to support recombinant human follicle-stimulating hormone (FSH)-induced follicular development in LH- and FSH-deficient anovulatory women: a dose-finding study. J Clin Endocrinol Metab. (1998) 83:1507–14. 10.1210/jc.83.5.1507 9589647

[126] Burgues S. The effectiveness and safety of recombinant human LH to support follicular development induced by recombinant human FSH in WHO group I anovulation: evidence from a multicentre study in Spain. Hum Reprod. (2001) 16:2525–32. 10.1093/humrep/16.12.2525 11726569

[127] De Moustier B Brinsden P Bungum L Fisch B Pinkstone S Warne D 0-158. The effects of combined treatment of recombinant (r)FSH and rLH in ratios 1:1, 2:1 and 3:1 in women. aged 38–42 years undergoing IVF-ICSI treatment. Hum Reprod. (2002) 17(Suppl 1):55 10.1093/humrep/17.suppl_1.54

[128] Humaidan P Chin W Rogoff D D'Hooghe T Longobardi S Hubbard J Efficacy and safety of follitropin alfa/lutropin alfa in ART: a randomized controlled trial in poor ovarian responders. Hum Reprod. (2017) 32:1537–8. 10.1093/humrep/dex208 PMC5946864 28541398

[129] Humaidan P Chin W Rogoff D D'Hooghe T Longobardi S Hubbard J Efficacy and safety of follitropin alfa/lutropin alfa in ART: a randomized controlled trial in poor ovarian responders. Hum Reprod. (2017) 32:544–55. 10.1093/humrep/dew360 28137754 PMC5850777

[130] Lehert P Chin W Schertz J D'Hooghe T Alviggi C Humaidan P. Predicting live birth for poor ovarian responders: the PROsPeR concept. Reprod Biomed Online. (2018) 37:43–52. 10.1016/j.rbmo.2018.03.013 29731240

[131] Alviggi C Conforti A Esteves SC Andersen CY Bosch E Buhler K. Recombinant luteinizing hormone supplementation in assisted reproductive technology: a systematic review. Fertil Steril. (2018) 109:644–64. 10.1016/j.fertnstert.2018.01.003 29653717

[132] Conforti A Esteves SC Di Rella F Strina I De Rosa P Fiorenza A The role of recombinant LH in women with hypo-response to controlled ovarian stimulation: a systematic review and meta-analysis. Reprod Biol Endocrinol. (2019) 17:18 10.1186/s12958-019-0460-4 30728019 PMC6366097

[133] Lehert P Kolibianakis EM Venetis CA Schertz J Saunders H Arriagada P. Recombinant human follicle-stimulating hormone (r-hFSH) plus recombinant luteinizing hormone versus r-hFSH alone for ovarian stimulation during assisted reproductive technology: systematic review and meta-analysis. Reprod Biol Endocrinol. (2014) 12:17. 10.1186/1477-7827-12-17 24555766 PMC4015269

[134] Mochtar MH Danhof NA Ayeleke RO Van der Veen F van Wely M. Recombinant luteinizing hormone (rLH) and recombinant follicle stimulating hormone (rFSH) for ovarian stimulation in IVF/ICSI cycles. Cochrane Database Syst Rev. (2017) 5:CD005070. 10.1002/14651858.CD005070.pub3 28537052 PMC6481753

[135] Santi D Casarini L Alviggi C Simoni M Efficacy of follicle-stimulating hormone (FSH) alone, FSH + luteinizing hormone, human menopausal gonadotropin or FSH + human chorionic gonadotropin on assisted reproductive technology outcomes in the personalized medicine era: a meta-analysis. Front Endocrinol. (2017) 8:114 10.3389/fendo.2017.00114 PMC5451514 28620352

[136] Braakhekke M Kamphuis EI Dancet EA Mol F van der Veen F Mol BW. Ongoing pregnancy qualifies best as the primary outcome measure of choice in trials in reproductive medicine: an opinion paper. Fertil Steril. (2014) 101:1203–4. 10.1016/j.fertnstert.2014.03.047 24786739

[137] Clarke JF van Rumste MM Farquhar CM Johnson NP Mol BW Herbison P. Measuring outcomes in fertility trials: can we rely on clinical pregnancy rates? Fertil Steril. (2010) 94:1647–51. 10.1016/j.fertnstert.2009.11.018 20056216

[138] Martins WP Niederberger C Nastri CO Racowsky C. Making evidence-based decisions in reproductive medicine. Fertil Steril. (2018) 110:1227–30. 10.1016/j.fertnstert.2018.08.010 30503110

[139] Mol BW Bossuyt PM Sunkara SK Garcia Velasco JA Venetis C Sakkas D Personalized ovarian stimulation for ART: study design considerations to move from hype to added value for patients. Fertil Steril. (2018) 109:968–79. 10.1016/j.fertnstert.2018.04.037 29935655

[140] Alviggi C Conforti A Esteves SC Vallone R Venturella R Staiano S. Understanding ovarian hypo-response to exogenous gonadotropin in ovarian stimulation and its new proposed marker-the follicle-to-oocyte (FOI) index. Front Endocrinol. (2018) 9:589. 10.3389/fendo.2018.00589 30386293 PMC6199413

[141] Gerrits MG Kramer H el Galta R van Beerendonk G Hanssen R Abd-Elaziz K. Oral follicle-stimulating hormone agonist tested in healthy young women of reproductive age failed to demonstrate effect on follicular development but affected thyroid function. Fertil Steril. (2016) 105:1056–62 e4. 10.1016/j.fertnstert.2015.12.017 26769303

[142] Abbotts C Salgado-Braga C Audibert-Gros C. A redesigned follitropin alfa pen injector for infertility: results of a market research study. Patient Prefer Adherence. (2011) 5:315–31. 10.2147/PPA.S21421 21792303 PMC3140313

[143] Schertz J Worton H. Nurse evaluation of the redesigned fertility pen injector: a questionnaire-based observational survey. Expert Opin Drug Deliv. (2018) 15:435–42. 10.1080/17425247.2018.1450386 29521156

[144] Schertz J Worton H. Patient evaluation of the redesigned follitropin alfa pen injector. Expert Opin Drug Deliv. (2017) 14:473–81. 10.1080/17425247.2017.1289174 28140682

